# Neutron resonance absorption imaging of simulated high-level radioactive waste in borosilicate glass

**DOI:** 10.1038/s41598-023-37157-2

**Published:** 2023-06-21

**Authors:** Y. Oba, R. Motokawa, K. Kaneko, T. Nagai, Y. Tsuchikawa, T. Shinohara, J. D. Parker, Y. Okamoto

**Affiliations:** 1 Materials Sciences Research Center, Japan Atomic Energy Agency, Tokai, Ibaraki 319-1195 Japan; 2 Present Address: Department of Mechanical Engineering, Toyohashi University of Technology, Toyohashi, 441-8580 Japan; 3 TRP Decommissioning Center, Japan Atomic Energy Agency, Tokai, Ibaraki 319-1194 Japan; 4 J-PARC Center, Japan Atomic Energy Agency, Tokai, Ibaraki 319-1195 Japan; 5 Neutron R&D Division, Comprehensive Research Organization for Science and Society (CROSS), Tokai, Ibaraki 319-1106 Japan

**Keywords:** Nuclear waste, Characterization and analytical techniques, Imaging techniques

## Abstract

We performed a preliminary study of neutron resonance absorption imaging to investigate the spatial distribution of constituent elements in borosilicate glasses containing simulated high-level radioactive waste, in which elemental inhomogeneities affect the physical and chemical stabilities of the glass. Dips generated by the resonance absorptions of Rh, Pd, Na, Gd, Cs, and Sm were observed in the neutron transmission spectra of the glass samples. The spatial distributions of these elements were obtained from the neutron transmission images at the resonance energies. The distributions of Rh and Pd visualized the sedimentation of these platinum group elements. In contrast, the lanthanides (Gd and Sm) and Cs were uniformly dispersed. These results show that neutron resonance absorption imaging is a promising tool for characterizing borosilicate glasses and investigating the vitrification mechanism of high-level radioactive waste.

## Introduction

Spent nuclear fuel reprocessing is key for the effective use of nuclear power. A solvent extraction method called the PUREX process is usually used in reprocessing to separate uranium and plutonium compounds from the spent nuclear fuel^1–3^. One problem with the PUREX process is that it also creates a large amount of high-level radioactive liquid waste (HLLW) containing fission products^4,5^. To avoid the migration of these products into the natural environment after geological disposal, HLLW is stabilized by a vitrification technique using borosilicate glass, in which the fission products are present as oxides^6–13^. Therefore, the stability of the vitrified high-level radioactive waste in the borosilicate glass is vital for ensuring long-term safety.

In the borosilicate glass, inhomogeneities, such as crystallized precipitates, affect its various physical and chemical properties and often cause instability. This behavior is well understood for platinum group elements, which are likely to be accumulated as crystallized precipitates at the bottom of melter during vitrification^14–21^. These precipitates increase the electroconductivity and viscosity of the waste-containing borosilicate glass melt and may disrupt the vitrification process.

These previous studies^14–21^ mentioned above have demonstrated that understanding the formation of elemental inhomogeneities is crucial in improving the stability of borosilicate glass containing high-level waste. Therefore, experimental observation is needed to clarify the spatial distribution of the constituent elements in the waste-containing borosilicate glass. Neutron imaging is useful for visualizing the elemental distributions because it has high penetration power, even for heavy elements. For example, neutron imaging has been used to observe the melting and solidification behaviors of a Pb-Bi alloy sealed in stainless-steel vessels^22–24^. In addition, the recent development of neutron resonance absorption imaging has enabled elemental mapping in samples^25–31^. The previous studies reported a wide variety of detectable elements by this technique: Ag, I^25^, Ta^26,28,29^, Na, Mn, Co, Rh, Cd, In, Xe, Cs, Sm, Eu, Dy, Er, Tm, Hf, W, Re, Ir, Au^27^, Cu^28,31^, and Zn^31^. Compared to other experimental techniques such as fluorescent X-ray spectroscopy, the neutron resonance absorption imaging can easily observe elemental distributions of thick samples even in furnaces or other sample environments^26,29^. In reality, practical vitrification plants equip with very large melters so that even neutrons hardly penetrate the melter filled with the waste-containing glass^4,11,32–35^. For example, the melter used for AVM (Atelier de Vitrification Marcoule) process has a working volume of about 100 L, which correspond to about 200 kg of waste-containing glass^4,34,35^. The U. S. government is constructing a latest plant known as WTP (Waste Treatment and Immobilization Plant) having two melters with the melt pool surface area of 3.75 m^2^^11^. Besides, the neutron resonance absorption imaging experiments require a pulsed neutron source, which is usually a particle accelerator and difficult to be installed near such practical vitrification plants. However, this technique is still valuable to laboratory-scale experiments^10,14,16,19,20^, which have produced meaningful results so far as basic researches about the vitrification behavior using a small amount of the simulated waste glass less than a few cm. In particular, this technique has potential for in situ experiments during the vitrification process. For example, neutron transmission at the neutron energy of 1 eV is 99% for Pt with the thickness of 1 mm, which is a general material of crucibles for the laboratory-scale experiments^36^. This is sufficiently high as background contribution for the neutron resonance absorption imaging. The high penetration power of neutrons also will allow three-dimensional elemental mapping via tomographic measurements and reconstruction^26,31^. However, there are only a few applications because this is a state-of-the-art technology owing to the development of neutron counting detectors with high temporal and spatial resolutions^29^.

In this study, neutron resonance absorption imaging is used to characterize the spatial distribution of the constituent elements and detect the inhomogeneities in borosilicate glass containing simulated high-level waste. Although the detectable elements were investigated in the previous studies^25–31^, the detection limit of each element can be affected by the absorption contributions of the other elements. In particular, borosilicate glasses often suffer from the large absorption contribution of boron in the neutron energy range used for the conventional neutron scattering experiments^36^. While the neutron resonance absorption image generally measures a higher neutron energy range, in which the absorption contribution of boron becomes smaller, the borosilicate glasses contain a large amount of boron. Therefore, a preliminary experiment is performed in this study. We clarify the rich potential of the neutron resonance absorption imaging. The results of this study will help to reveal the vitrification mechanism of HLLW and improve the stability of waste-containing borosilicate glass.

## Experiment

**Figure 1:**
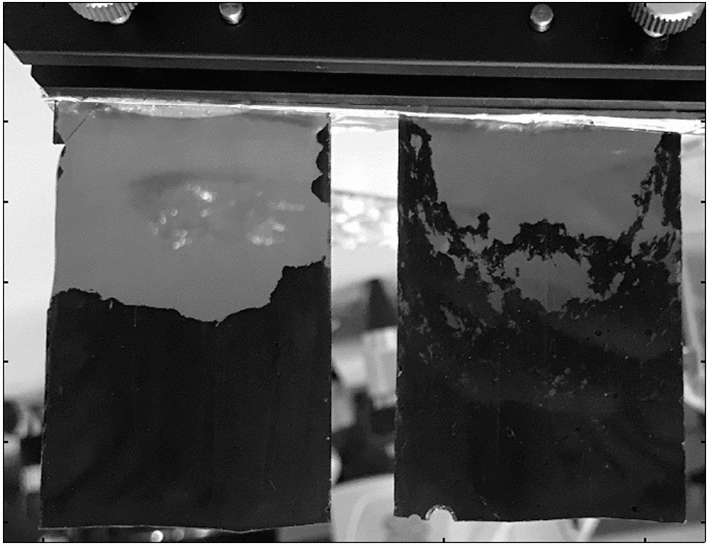
Photograph of the glass samples. The left and right plates are samples 1 and 2, respectively.

The samples were simulated waste glasses. The chemical compositions of the samples are listed in Table 1. Sample 1 was prepared to characterize mainly the behavior of platinum group elements. Sample 2 was composed of the simulated high-level waste similar to the previous studies in Japan^15,20,37^, which is composed of basically more than twenty of elements including Na, platinum group elements, lanthanides, and Cs. Except Na and the platinum group elements, the chemical compositions of the simulated high-level waste were less than a few %. The glass frits of these simulated waste glasses were loaded into Pt containers placed in alumina crucibles and held at 1100 °C for 720 h for sample 1 and 240 h for sample 2. Since this is a preliminary experiment, the samples should have a simple shape with a uniform thickness for simplicity. In addition, thin samples are better to estimate the detection limit. Therefore, the glasses were then cut vertically into 1-mm-thick plates to observe the cross sections (Fig. 1). Black precipitates were visible at the bottom of the samples. These precipitates are formed due to the segregation of the oxides and metals of the platinum group elements and contribute to the black color with light absorption^14–21^.

**Table 1:** Chemical compositions of simulated waste glass samples. The corresponding values in mole percent were calculated with the assumption that there is no other element.

Sample no.	SiO_2_	B_2_O_3_	CaO	Al_2_O_3_	ZnO	Li_2_O	Na_2_O	ZrO_2_	RuO_2_	Rh_2_O_3_	PdO	LnO_*x*_ + Cs_2_O + others
1 (wt %)	49.0	13.6	2.9	5.2	3.2	3.2	8.4	2.5	3.3	0.85	1.9	Balance
(mol %)	55.6	13.3	3.5	3.5	2.6	7.2	9.3	1.4	1.7	0.23	1.1	
2 (wt %)	45.0	12.0	2.9	3.2	2.9	2.9	9.3	1.6	1.7	0.17	0.51	Balance
(mol %)	56.6	13.0	3.9	2.4	2.7	7.3	11	0.98	0.97	0.05	0.32	

*Ln* lanthanides (Gd and Sm).

The neutron transmission imaging measurements were performed at the neutron imaging apparatus RADEN (BL22) of the Materials and Life Science Experimental Facility (MLF), the Japan Proton Accelerator Research Complex (J-PARC)^31^. The operation beam power was 500 kW for the MLF. The basic parameters were summarized in Ref.^31^. A two-dimensional micropattern neutron detector (*µ*NID) was used to obtain the neutron transmission images^38^. The bin size and effective area of the detector were 0.25 × 0.25 mm and 100 × 100 mm (height × width), respectively. The neutron energy was calibrated from the resonance absorption dips of Cd, which was placed in the neutron beam, and Na included in the samples. The neutron transmissions were calculated as the ratio of the neutron intensities measured with the samples to those without the samples. Both the neutron intensities were normalized by the number of the incident neutron pulses, which were 597,240 (with the samples) and 348,053 pulses (without the samples) corresponding to 6.6 and 3.9 h, respectively. Background contributions including dark current noise and scattered neutrons from environment was ignored similar to previous work using the *µ*NID^38^. The uncertainties of neutron intensities were estimated based on the Poisson distribution, which provides that both the mean and variance are equal to the measured neutron counts and commonly used in the neutron scattering experiments and transmission analyses^39–42^. The standard errors of the neutron transmission were evaluated from the uncertainties of the neutron intensities based on the law of propagation.

## Results and discussion

**Figure 2:**
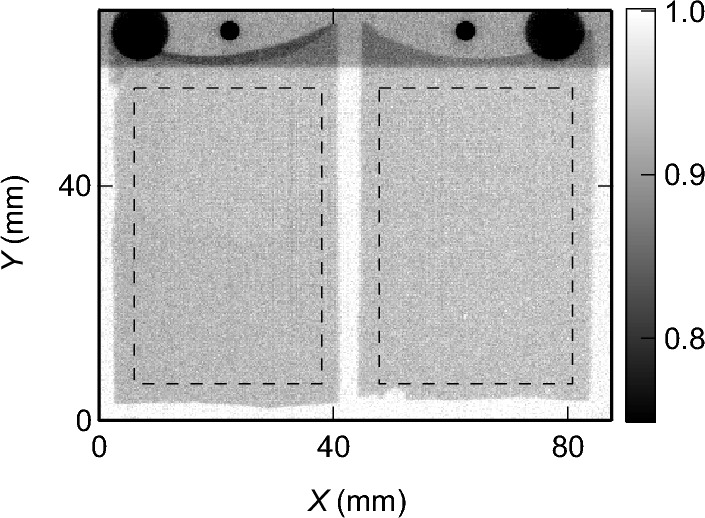
Neutron transmission images of the glass samples averaged in the neutron energy range between 0.5 eV and 10 keV. Dashed rectangles denote the area used to evaluate the neutron transmission spectra in Fig. 3. The top shows the shadow of the sample holder.

Figure 2 shows the neutron transmission image of the glass samples averaged in the neutron energy range of 0.5 eV to 10 keV. The image reflects the shapes of the glass plates. The values of the neutron transmission are almost uniform in the samples. The difference between the transparent and black regions that are visible in the photograph (Fig. 1) is indistinct in the neutron transmission images.

**Figure 3:**
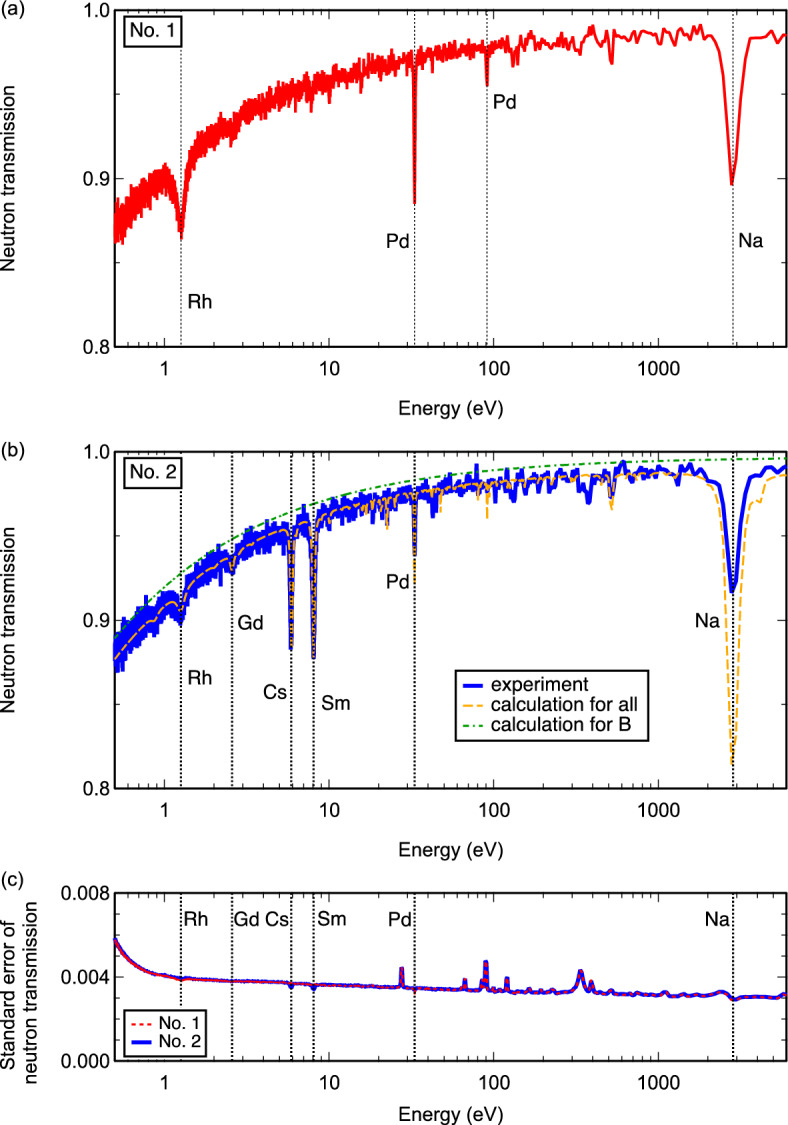
Neutron transmission spectra averaged over the entirety of (**a**) sample 1 and (**b**) sample 2. Vertical dotted lines indicate the reference values of the resonance energies^43^. (**c**) Standard errors of neutron transmission.

Figure 3 shows the neutron transmission spectra averaged over the entirety of samples 1 and 2 (dashed rectangles in Fig. 2). In both samples, the spectra are characterized by a base curve gently increasing from 0.87 at 0.5 eV to 0.99 at 10 keV. This gentle curve coincides with the characteristics of the neutron cross section of boron^43^. In addition, both the spectra have several dips caused by the resonance absorption of the constituent elements^25–31^. Similar to visible light, the neutron transmission follows the Beer-Lambert law^25,44^. Hence, the minimum neutron transmission of 0.87 at a thickness of 1 mm means that the same sample with a thickness of 10 mm would still have a neutron transmission of 0.25, which is enough for experimental observations, and demonstrates the high penetration power of neutrons. This indicates that the neutron resonance absorption imaging can be applied to the samples used in the laboratory-scale experiments, which typically use the simulated waste glass less than a few cm^10,14,16,19,20^.

Based on the standard nuclear data library, JENDL-4.0^43^, the elements corresponding to the dips were determined. Although the resonance energies depend on nuclides rather than elements, no enriched isotope was used for the current samples. Therefore, the weighted-average data with natural abundance ratios were referred to as the resonance absorption of each natural element in the following sections for simplicity. The apparent dips appear at 1.3, 33, 91, and 2850 eV in sample 1 (Fig. 3a). Among the elements listed in Table 1, only Rh has a resonance absorption at 1.3 eV. Similarly, the relatively sharp dips at 33 and 91 eV are attributed to Pd. The broad dip at 2850 eV results from the resonance absorption of Na. In sample 2, the dips are detected at 1.3, 2.6, 5.9, 8.0, 33, and 2850 eV (Fig. 3b). The dips at 1.3, 33, and 2850 eV are also observed in sample 1 and are attributed to Rh, Pd, and Na, respectively. Although the dip at 91 eV for Pd is not seen in sample 2, this is probably because of the smaller amount of Pd in sample 2 than in sample 1. The resonance absorption of Ru is not found even though its content is higher than those of the other platinum group elements (Rh and Pd) in both the samples. This is explained by the smaller neutron cross section of Ru, in which the maximum is 3 × 10^2^ barns (at 625 eV). In contrast, the maxima are 5 × 10^3^ barns (at 1.3 eV) for Rh and 7 × 10^3^ barns (at 33 eV) for Pd between 0.5 eV and 10 keV^43^. The dips at 2.6 and 5.9 eV are assigned as Gd and Cs. The dip at 8.0 eV can be explained by both Sm and Gd. However, for Gd, the dip at 8.0 eV must be smaller than that at 2.6 eV. Therefore, the main contribution to the resonance energy of 8.0 eV is determined as Sm.

The heights of the resonance absorption dips reflect the contents of the corresponding elements. Hence, the neutron resonance absorption analysis can give information about chemical composition in principle^27,28,30,31^. Based on the Beer-Lambert law, neutron transmission *T* including the resonance absorption is described as

1 $$T=\mathrm{exp}\left(-t\sum_{i}{\sigma }_{i}{n}_{i}\right),$$

where *t*, *σ*_*i*_, and *n*_*i*_ denote the thickness of the sample, the neutron cross section, and the number density of the *i*th element (weighted average of isotopes with the natural abundance ratio). Each element has a unique set of resonance energies in *σ*_*i*_. Given that sample 2 contains Gd, Cs, and Sm as well as the elements listed in Table 1, the observed resonance energies in sample 2 are explained well by the Eq. (1) (dashed line in Fig. 3b). Here, the contribution of oxygen is ignored because it is tiny compared with the other elements^43^. The values of *n*_*i*_ are expressed as *n*_*i*_ = *ax*_*i*_, where *x*_*i*_ is the mole fraction and *a* is the average atomic density in the samples and functions as a scale factor in the present analysis. The values in Table 1 are used as the fixed parameters for *x*_*i*_, except for Gd, Cs, and Sm. Consequently, the heights of the dips for Gd, Cs, and Sm can be represented with *x*_*i*_ of 0.2, 0.2, and 0.04 mol% for Gd_2_O_3_, Cs_2_O, and Sm_2_O_3_, respectively. These values are consistent with the previous studies^15,20,37^. The calculated contribution of B was also plotted in Fig. 3b. This clearly reveals that the gentle curve from 0.87 at 0.5 eV to 0.99 at 10 keV is mainly attributed to B. The calculated height for Na does not match the observed height well. This suggests that the statistical accuracy and energy resolution of the current experiment were probably insufficient in this high-energy range. A similar discussion about this problem was reported in a previous study^27^. Another possibility is a background signal, which can be considerable in a higher neutron energy range and cause the underestimation of the neutron transmission^29^.

From the standard error of the neutron transmission, the detection limits are estimated for the observed elements. As shown in Fig. 3c, the magnitudes of the standard errors are around 0.004 between 0.5 and 6000 eV for both the samples. If the observable minimum heights of the neutron resonance dips are defined as the magnitudes of the standard errors at the corresponding resonance energies, the detection limits can be estimated. Table 2 summarizes the concentration equivalent to the standard errors of the neutron transmission for the individual oxides. The detection limits can be regarded as two to three times the equivalent concentrations. For Gd_2_O_3_, the content of 0.2 mol% already approximates to the detection limit, which means the low detectability of the neutron resonance absorption analysis for Gd. The detectability is also low for Na as shown by the high equivalent concentrations of 0.3 and 0.5 mol% in samples 1 and 2, respectively. In this case, the large amount of Na allows the observation of the neutron resonance absorption. The other values of the equivalent concentrations are below 0.1 mol%. In particular, the detection limit for Sm is significantly lower than those for the other elements resulting from the large neutron cross section of 5 × 10^4^ barns at 8.0 eV^27,43^. This means that Sm can be a useful indicator to characterize the behavior of lanthanides. Although the gradual decrease toward the lower neutron energy may deteriorate the detectability of the dips at the low resonance energies, the detection limit of Rh is still comparable to that of Pd.

**Table 2:** Concentrations of oxides equivalent to the standard error obtained from the neutron transmission.

Sample No	Rh_2_O_3_	Gd_2_O_3_	Cs_2_O	Sm_2_O_3_	PdO	Na_2_O
1 (wt %)	0.09				0.08	0.3
(mol%)	0.02				0.04	0.3
2 (wt %)	0.05	0.4	0.04	0.008	0.05	0.4
(mol%)	0.01	0.08	0.01	0.002	0.03	0.5

**Figure 4:**
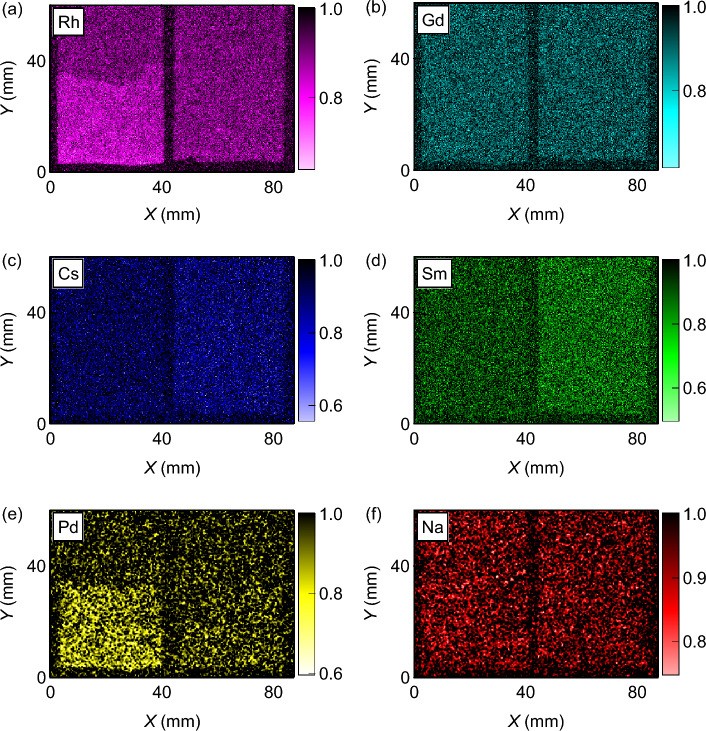
Neutron transmission images of the glass samples at the resonance energies of (**a**) Rh, (**b**) Gd, (**c**) Cs, (**d**) Sm, (**e**) Pd, and (**f**) Na. The shadow of the sample holder is omitted for simplicity.

The neutron transmission image at the resonance energy can visualize the spatial distribution of the corresponding element as the sharp decrease of the neutron transmission. Figure 4a–f show the neutron transmission images at the resonance energies of the individual elements. Each transmission image was obtained as the average within the full width at half maximum (FWHM) around the corresponding resonance energy. For Pd, the dip at 33 eV was analyzed. The behavior of the platinum group elements should be observed more clearly in sample 1 than in sample 2 because of the higher Rh and Pd contents. In Fig. 4a, Rh is concentrated at the bottom of sample 1, shown as low transmission regions. The contour of this region agrees well with the black region shown in Fig. 1, indicating that Rh is mainly contained in the precipitates. The distribution of Rh in sample 2 is unclear, probably because of the lower content. Similar behavior is observed in the distribution of Pd. These results are consistent with the conventional understanding that the platinum group elements cause sedimentation during heating^14–21^. In contrast, sample 2 is suitable to observe the behavior of Cs and lanthanides (Gd and Sm) as seen in Fig. 3b. These elements are uniformly dispersed throughout sample 2, indicating that the borosilicate glass can hold those elements well. The image of Na shows no major inhomogeneity and suggests that glass network modifiers are uniformly dispersed.

These results demonstrate that neutron resonance absorption imaging is a powerful method for characterizing the spatial distribution of constituent elements, including platinum group elements, Cs, and lanthanides, in waste-containing borosilicate glass. In particular, the high penetration power of neutrons will allow in situ observation through furnaces or crucibles during the vitrification process, whereas most conventional structural analyses use specimens that are cooled after vitrification. This will provide us with opportunities to study the dynamics of the vitrification process experimentally.

## Conclusion

We performed neutron resonance absorption imaging to investigate the spatial distribution of constituent elements in the borosilicate glasses containing simulated high-level waste. Resonance absorption dips for Rh, Pd, Na, Gd, Cs, and Sm were detected in the neutron transmission spectra and the average contents of Gd, Cs, and Sm were estimated from the heights of the dips. The neutron transmission images at those resonance energies revealed the distributions of corresponding elements. The images confirmed that platinum group elements cause the sedimentation, whereas lanthanides and Cs were held uniformly in the borosilicate glass. These results indicate that neutron resonance absorption imaging has the potential to advance the study of the vitrification mechanism.

## Data Availability

The datasets generated during and/or analyzed during the current study are available from the corresponding author on reasonable request.
